# Domestic Economy in the Isolation Hospital

**Published:** 1916-02-12

**Authors:** 


### DOMESTIC ECONOMY IN THE ISOLATION HOSPITAL.

### An Example of Prudent Housekeeping.

THE problem to be solved is as follows. Given an institution with a widely varying number of patients, the wards sometimes nearly all empty, and then again overcrowded during an epidemic, with a staff varying in numbers according to the increase in patients, with a system of accounts in which not merely the food for patients and staff is reckoned under one heading, but domestic expenses also are included, how is the matron to demonstrate to her committee that due economy has been observed when an increase of over £200 appears on "provisions and domestic expenses" in 1915 as compared with 1914?

Commencing with the food, we find that in 1915 there was an average of 9.3 occupied beds, as compared with an average of just under six in 1914. The number of days' maintenance for patients was 3,398 in 1915 and 2,147 in 1914. On a given day two patients out of twelve were on convalescent diet. But it is not so important a matter as it may seem to disentangle the patients on low diet from those on convalescent diet. For the majority of the patients were typhoids, and their diet was very nearly as costly as that of the convalescents. It consisted of two quarts of milk, 7d.; one pint of Benger, 3d.; one pint of soup, 2d.; total, 1s. a day. To reckon the all-round daily cost of the patients' food at 1s. a head is probably not far from the mark. Many of them are workers in big blast-furnaces, and they need very good nourishment and stimulant (probably beer or porter is allowed, but this is not mentioned) to build up their strength in convalescence. For this reason we consider that an average of 1s. a day is not excessive taking all the diets together. In 1915, then, the cost of the patients' diet may be reckoned at £170, and in 1914 at £107.

Turning to the staff we find a household consisting of the matron, two probationers, a cook-caretaker and her gardener-husband, a kitchen- or house-maid, two ward-maids, and two laundry-maids, the four latter being out-workers. During 1915, owing to epidemics, the staff was increased by six nurses engaged for various periods. Collectively these nurses were boarded for 384 days, and as additional service was engaged also during this period it would be reasonable to reckon the extra maintenance as equivalent to that of two extra persons in the year. Unluckily no information has been vouchsafed as to whether the ward-maids and laundry-maids received board or no. We assume, on the analogy of other institutions of a like character, that the ward-maids did and the laundry-maids did not receive board. We have, therefore, an average of eight persons boarded in 1914, and the equivalent of ten in 1915.

As regards the rate at which the staff is boarded, we should not expect in a household of this description, with varying numbers and extra help engaged from time to time, that a lower average could be maintained than from 8s. to 10s. a week per head. Estimating the cost at the middle figure, 9s. a week, the yearly average per head would be £23. To keep the expenses at this figure would require good management, for there is no doubt that the boarding of extra nurses entails more expense than that of the ordinary staff. The cost of the food for the nursing staff cannot be separated from that of the domestic staff. Collectively, the cost of food for the household accounts for a total of £184 in 1914 as against £230 in 1915.

We now come to an important consideration. Prices had risen very considerably between the year ending March 1914 and that ending March 1915. Were it not for the fact that the extra amount paid to cover higher prices on contract goods has been specified, the above calculations could have no value. But this vital piece of information has been accorded us. We find that higher prices account for no less than £79 extra in 1915, or 15 per cent. Thus we are able to estimate with some reasonable confidence the rate of expenditure during the two contrasted years. The figures work out as follows:—

| | 1915 | 1914 |
|---|---|---|
| Provisions for patients at 1s. per head per diem ... ... ... ... ... | £170 | £107 |
| Provisions for staff at 9s. per head per week ... ... ... ... ... | 230 | 184 |
| Higher prices for contract goods ... | 79 | — |
| Balance for domestic expenses, cleaning materials, etc. ... ... ... ... | 31 | 12 |
| Totals representing actual expenditure | £510 | £303 |

It has to be borne in mind that the 1915 year was one of bad epidemics. The extra amount spent on "cleaning materials," whatever that may include, is thus thoroughly accounted for.

It will be seen from the foregoing figures that the matron can be fully vindicated from any breach of economy, and we have little doubt that a really close investigation of the accounts would bear out the conclusions we have arrived at with the slender data at our command. The conclusions would not be materially affected were the amount expended on material other than provisions a little more or less.

